# The Levels of Polycyclic Aromatic Hydrocarbons and Their Derivatives in Plasma and Their Effect on Mitochondrial DNA Methylation in the Oilfield Workers

**DOI:** 10.3390/toxics11050466

**Published:** 2023-05-17

**Authors:** Yaning Jia, Weixia Li, Yanlin Li, Lei Zhao, Chenguang Li, Lei Wang, Junkai Fang, Shanjun Song, Yaqin Ji, Tao Fang, Jing Zhang, Liqiong Guo, Penghui Li

**Affiliations:** 1 Institute of Disaster and Emergency Medicine, Tianjin University, Tianjin 300072, China; jiayaning@tju.edu.cn (Y.J.); li15294991853@163.com (W.L.); zhaolei0130@163.com (L.Z.);; 2 Tianjin Fourth Central Hospital, Tianjin 300140, China; 3 Wenzhou Safety (Emergency) Institute, Tianjin University, Wenzhou 325000, China; 4 Tianjin Boshengyuan Environmental Technology Center, Tianjin 300381, China; liyanlin913@hotmail.com; 5 School of Environmental Science and Safety Engineering, Tianjin University of Technology, Tianjin 300384, China; chenguang00513@163.com (C.L.);; 6 Tianjin Key Laboratory of Hazardous Waste Safety Disposal and Recycling Technology, Tianjin 300384, China; 7 Hebei Research Center for Geoanalysis, Baoding 071000, China; wanglei8812@126.com; 8 Tianjin Institute of Medical & Pharmaceutical Sciences, Tianjin 300070, China; mephist111@163.com; 9 National Institute of Metrology, Beijing 100029, China; songsj@nim.ac.cn; 10 College of Environmental Science and Engineering, Nankai University, Tianjin 300071, China

**Keywords:** PAHs, PAH derivatives, exposure, plasma, methylation

## Abstract

This study focuses on the components and levels of polycyclic aromatic hydrocarbons (PAHs) and their derivatives (MPAHs and OPAHs) in plasma samples from 19 oil workers, pre- and post-workshift, and their exposure–response relationship with mitochondrial DNA (mtDNA) methylation. PAH, MPAH, OPAH, and platelet mtDNA methylation levels were determined using a gas chromatograph mass spectrometer (GC-MS) and a pyrosequencing protocol, respectively. The total plasma concentrations of PAHs in mean value were, respectively, 31.4 ng/mL and 48.6 ng/mL in pre- and post-workshift, and Phe was the most abundant (13.3 ng/mL in pre-workshift and 22.1 ng/mL in post-workshift, mean value). The mean values of total concentrations of MPAHs and OPAHs in the pre-workshift were 2.7 ng/mL and 7.2 ng/mL, while in the post-workshift, they were 4.5 ng/mL and 8.7 ng/mL, respectively. The differences in the mean *MT-COX1*, *MT-COX2*, and *MT-COX3* methylation levels between pre- and post-workshift were 2.36%, 5.34%, and 0.56%. Significant (*p* < 0.05) exposure–response relationships were found between PAHs and mtDNA methylation in the plasma of workers; exposure to Anthracene (Ant) could induce the up-regulation of the methylation of *MT-COX1* (*β* = 0.831, SD = 0.105, *p* < 0.05), and exposure to Fluorene (Flo) and Phenanthrene (Phe) could induce the up-regulation of methylation of *MT-COX3* (*β* = 0.115, SD = 0.042, *p* < 0.05 and *β* = 0.036, SD = 0.015, *p* < 0.05, respectively). The results indicated that exposure to PAHs was an independent factor influencing mtDNA methylation.

## 1. Introduction

Growing concern over persistent organic pollutants (POPs) has been witnessed in the past years [1]. Polycyclic aromatic hydrocarbons (PAHs), which are compounds with two or more fused benzene rings, are one of the most common POPs in nature [2,3]. Studies have found their widespread distribution in different environmental matrices, such as indoor environments (houses, schools, cars, etc.) and sediments from an open-pit mining area [4,5]. It has been well established that PAHs have detrimental biological effects, such as mutagenicity, toxicity, and carcinogenicity, posing a serious threat to human health [6,7]. A total of 16 unsubstituted PAHs, consequently, have been listed as priority control pollutants by the US Environmental Protection Agency (US EPA). Additionally, several PAHs (Naphthalene, Fluorene, Phenanthrene, Anthracene, etc.) were added to the black list or gray list as priority pollutants in many other countries including China [8,9]. Attention has been focused on PAH derivatives such as methylated PAHs (MPAHs) and oxygenated PAHs (OPAHs) in recent years, since their toxicity may be the same or higher than the parent PAH [10].

PAHs and their derivatives (MPAHs and OPAHs) are ubiquitous in the environment due to their multiple sources. The main source of PAHs is the incomplete combustion of organic matter caused by natural processes or anthropogenic activities, and at present, human activities are reported to be the main source of emissions, including emissions from the burning of bio-organic matter, automobile exhaust, oil extraction, industrial use, and so on. Similarly to their parent compounds, MPAHs and OPAHs can originate from petrogenic or pyrogenic sources [11]. In addition, MPAHs and OPAHs can also generate from PAHs through the transformation and metabolism of their parent PAHs [12]. The main exposure pathways of PAHs and their derivatives for the human body include inhalation, ingestion, and dermal contact [13]. The exogenous chemicals of pollutants are first absorbed by the blood and then distributed, metabolized, and excreted through the blood system, which can then be transported to a depot or target organ [14,15]. The effects of exposure to PAHs on human health have been demonstrated by many studies. For instance, a variety of diseases can be induced by PAHs, such as cataracts, kidney and liver damage, jaundice, lung cancer, skin cancer, and bladder cancer [7,16]. As of now, most investigations into PAHs and PAH derivatives have mainly focused on their concentrations and sources in the environment; however, limited information has been reported for cumulative exposure levels in human plasma and their relationship with health risks.

Many studies have recently indicated that epigenetic mechanisms represent a novel thought, which may help researchers understand the interaction between pollutants and the genomes and their effects on chronic diseases. Experimental models and studies have shown that with environmental exposure, DNA methylation is susceptible to change [17,18]. Studies have shown that airborne pollutants can induce DNA methylation in peripheral blood nuclear DNA, and there are associations between IL-12 and p53 DNA methylation and exposure to PAHs [19,20]. In recent studies, it has been shown that, due to the lack of protective histones, mitochondrial DNA (mtDNA) is more sensitive to the damages caused by exogenous reactive oxygen sources than nuclear DNA [21]. In mitochondria, small mtDNA molecules exist independently from nuclear DNA. It is a separate epigenetic machinery that generates mtDNA methylation and is the primary source of oxidative stress generation in response to exogenous environments [22]. The study on mtDNA methylation aimed to reflect epigenomic reprogramming induced by environmental exposure and the potential risk of future diseases [23,24]. Although there has been increasing focus on mtDNA methylation with breakthroughs achieved, epigenetic mechanisms in mtDNA induced by exposure to PAHs and their derivatives (MPAHs and OPAHs) remain limited, particularly in human investigations.

It is known that large quantities of PAHs and their derivatives will be released during the oil extraction process [25]. Therefore, for oil workers, the exposure levels and health risks are believed to be much higher than those for normal populations. In this study, 38 blood samples were collected from 19 oil-production workers in the pre- and post-workshifts, respectively. Based on measuring both PAHs in plasma and *MT-COX* gene loci, the main objectives of this study include the following: (1) to determine the components and concentration of PAHs, MPAHs, and OPAHs accumulated in human plasma in pre- and post-workshift; (2) to estimate mtDNA methylation levels in pre- and post-workshift; (3) to examine the extent to which individual PAH compounds are related to mtDNA methylation, and conduct a health risk assessment using mtDNA methylation as a biomarker.

## 2. Materials and Methods

### 2.1. Study Population and Sample Collection

A total of 19 Chinese workers who worked abroad in an oilfield in Iraq were enrolled in this study. Before sample collection, demographic data such as age, gender, height, weight, and length of service were collected by trained physicians and/or nurses. Blood samples were collected twice, both in China. The first collection took place before the workers went abroad (pre-workshift), and the second collection was conducted when they had returned to China after working in the field for 30 days (post-workshift). Dwelling times of these workers are shown in Table S1. Venous blood was collected from each subject at the hospital; the fasting blood was obtained in 2 mL EDTA vacuum blood collection tubes containing anticoagulation and then centrifuged at 1710 g for 10 min to separate the plasma. A volume of 1 mL of supernatant from each sample was transferred into a 1.8 mL cryo vial (KG2731, KIRGEN, Shanghai, China) using the pipettor, and the marked tubes were immediately stored temporarily at a −20 °C refrigerator. After sampling, all plasma samples were rapidly transferred to the laboratory and kept at −80 °C in the freezer prior to pretreatment and further analysis. All participants were informed in detail about the aim of the study, and a consent form was required for each subject. This project complies with the Human Medical Ethic Review Law of the Ministry of Health and the Declaration of Helsinki.

### 2.2. Determination of PAHs and PAH Derivatives in Plasma

#### 2.2.1. Pretreatment

Sample extraction and cleanup were performed as previously described with some modifications [26,27]. Briefly, 500 µL of plasma was placed into a 10 mL polypropylene centrifuge tube. Then, 1 mL of ammonium acetate solution (1 M), 0.25 mL of formic acid solution (1 M), and 1.2 mL of ultrapure water were added to denature proteins. The mixture was homogenized by ultrasonication for 10 min, following by centrifuging at 3040 g for 5 min. Cleanup of the extract was performed using solid phase extraction (SEP) cartridges (Waters OAsis HLB 6 cc (500 mg) LP Extraction Cartridge), which, before sample loading, were activated by 5 mL of n-hexane, 5 mL of dichloromethane, 5 mL of acetone, 5 mL of methanol, and 5 mL of ultrapure water, in order. After this step, the supernatant was spiked with 40 µL of surrogate standard (Phe-D_10_ and Chr-D_12_) and placed on top of the SPE cartridge. The cartridge was rinsed with 2 mL of diluted hydrochloric acid (0.1 M) and 2 mL of ultrapure water to remove strong polar impurities in the plasma. The elution for PAHs, MPAHs, and OPAHs was performed using 9 mL of mixture solvent of n-hexane/dichloromethane (*v*:*v* = 1:1). Then, under a gentle flow of nitrogen and redissolved with 500 µL of n-hexane, the eluted fractions were evaporated.

#### 2.2.2. GC–MS Analysis

PAHs, MPAHs, and OPAHs determination was performed using the gas chromatograph mass spectrometer system (GC-MS, SHIMADZU 2010, Kyoto, Japan) with DB-5MS capillary column (SHIMADZU; 30 × 0.25 mm, film thickness 0.25 μm). The 5 µL samples were injected in the pulsed splitless mode with an injection temperature of 270 °C. The column temperature was set at 65 °C for 0.5 min at first and raised to 130 °C at the rate of 15 °C min^−1^ for 0.5 min, to 220 °C at the rate of 9 °C min^−1^ for 1 min, to 240 °C at the rate of 7 °C min^−1^ for 1.5 min, to 320 °C at the rate of 15 °C min^−1^ for 5 min. The mass spectrometry conditions were set as electron ionization (EI) and selective ion monitoring (SIM) modes, with the ion source temperature at 230 °C. Finally, six PAHs, two MPAHs, and one OPAH were detected in the plasma samples. The monitored ions of all analytes and surrogate standards were summarized in Table S2 in the supporting information.

### 2.3. DNA Methylation Analysis

Plasma samples were collected according to the sequencing protocol and stored at −80 °C until later use. Then, the plasma samples were extracted for platelet mtDNA, and the methylation levels were detected by pyrosequencing protocol. By applying this method, which was developed by Andrea A. Baccarelli and Hyang min Byun’s team, subtle differences in methylation levels can be detected [28]. The main steps are as follows: bisulfite conversion of 1 µg of mtDNA using EZ DNA methylation kit (Zymo Research, Seattle, WA, USA); 1 µL of transformed DNA used for polymerase chain reaction (PCR) amplification by GoTaq hot start polymerase (Promega, Madison, WI, USA); methylation level in three specific *MT-COX* gene loci, i.e., the *COX1*, *COX2*, and *COX3* regions examined by bisulfite pyrosequencing. Details of the primers and thermocycling conditions are shown in Table 1.

### 2.4. Quality Assurance and Quality Control (QA and QC)

All instruments were calibrated each day under strict calibration standards. To ensure the accurate determination of PAHs, MPAHs, and OPAHs, a reagent blank consisting of 500 µL of n-hexane was included in every batch (5 samples) of plasma samples, and none of the analytes were detected in the blanks. To monitor the matrix effects as well as the extraction performance, surrogate standards were added to each sample. The average recoveries of the two surrogate standards from all the blood samples and blanks were between 87.6% and 183%, with relative standard deviation (RSD) less than 10% in matrix spikes. We defined the limit of detection (LOD) of targeted compounds at 3 times the signal-to-noise ratio, ranging from 0.40 to 2.00 ng/mL for PAHs and their derivatives.

Strict QA/QC procedures were performed during DNA methylation analysis. All equipment that had contact with samples was sterilized, non-pyrogenic, and DNAse/RNAse-free. We used methylated genomic DNA in pyrophosphate sequencing (samples with known methylation rate) as positive control and water blanks in polymerase chain reaction as negative control. Each DNA sample was analyzed on at least two runs by pyrosequencing.

### 2.5. Statistical Analysis

Age, length of service, and body mass index (BMI) were included in statistical analyses as covariates. The statistically significant differences in the concentrations of PAH, MPAH, OPAH, and DNA methylation levels among the pre-exposure and post-exposure groups were evaluated by the group square difference in random area adjusted for appropriate covariates. To determine the effect of environmental exposures, mixed-effect models were used to analyze the effects of PAHs, MPAHs, and OPAHs on mtDNA methylation levels while controlling for covariates. To indicate significant differences in data analysis, value of *p* < 0.05 was considered. SAS (version 9.4) was applied for statistical analysis.

## 3. Results

### 3.1. PAH, MPAH, and OPAH Concentration Levels

The individual values of all the components (PAHs, MPAHs, and OPAHs) detected are presented as the 25th percentile, 50th percentile (median), and 75th percentile, with detection frequencies, in Table 2. The detection frequencies of all the components were in the range of 0–100%. In both the pre- and post-workshift, the detection frequency of Dibf, Flo, Phe, Flu, and Pyr are all above 70%. The post-exposure levels of all the subjects were much higher than that of pre-exposure, where the median value of 44.0 ng/mL increased from 35.2 ng/mL.

### 3.2. mtDNA Methylation Levels

**Figure 1:**
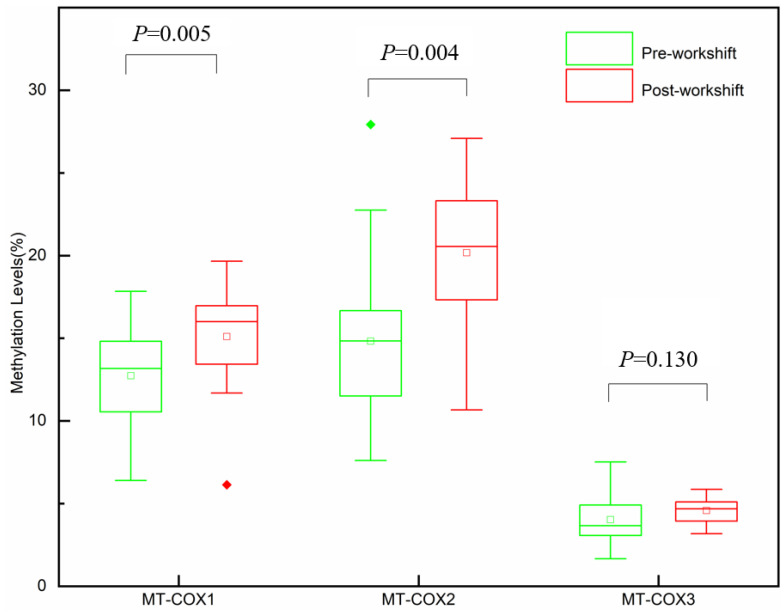
Distribution of percentage mtDNA methylation levels (%) in pre-workshift and post-workshift for *MT-COX1*, *MT-COX2*, and *MT-COX3*. The lower and upper limits of boxes represent the 25th to 75th percentiles, and the internal solid bars represents the median values. Three data points represent the minimum, mean, and maximum values from bottom to top, respectively. The whiskers range within 1.5 IQR.

Platelet mtDNA methylation was measured by bisulfite PCR combined with pyrosequencing pre- and post-workshift. The methylation levels of three specific *COX* gene loci, i.e., the *MT-COX1*, *MT-COX2,* and *MT-COX3* regions, were examined. The median methylation levels of *MT-COX* are shown in Table S3. *MT-COX1* and *MT-COX2* methylation levels with the post-workshift (mean = 15.10%, mean = 20.17%; respectively) were significantly (*p* < 0.05) higher than those of the pre-workshift (mean = 12.74%, mean = 14.83%; respectively). The difference in the mean *MT-COX1* and *MT-COX2* methylation levels between the pre-workshift and post-workshift was 2.36% and 5.34% (*p* < 0.05). In addition, no significant statistical differences were found in changes in the methylation level of *MT-COX3* between pre-workshift and post-workshift (Figure 1).

### 3.3. Association between mtDNA Methylation and Pollutant Exposure Levels

Among all the pollutants, Nap and Nap-1 were automatically excluded due to their low detection frequencies. The regression coefficient *β*, standard deviation, and corresponding *p*-value were obtained and are presented in Table 2.

For *MT-COX1*, we found that Ant could induce the up-regulation of the methylation level (*β* = 0.831, SD = 0.105, *p* < 0.05), illustrating that a one-unit increment of Ant could result in a 0.831% increase in the *MT-COX1* methylation levels. For *MT-COX3*, we found that Flo and Phe could induce the up-regulation of the methylation levels (*β* = 0.115, SD = 0.042, *p* < 0.05 and *β* = 0.036, SD = 0.015, *p* < 0.05; respectively), illustrating that a one-unit increment of Flo and Phe could result in a 0.115% and 0.036% increase in *MT-COX3* methylation levels, respectively. No association of PAHs and their derivatives with the methylation level of *MT-COX2* was found (Table 3).

## 4. Discussion

To the best of our knowledge, this study is the first to link the adverse effects and exposure–response relationship between mtDNA methylation and PAHs and their derivatives (MPAHs and OPAHs). To evaluate the effect of pollutant exposure in oilfields, the concentrations of PAHs and mtDNA methylation levels were examined pre- and post-workshift, respectively. The results showed that plasma pollutants and mtDNA methylation increased post-workshift. Additionally, the exposure–response relationship between PAHs and MT-DNA methylation was observed, indicating that exposure to PAHs can induce the up-regulation of the mtDNA methylation.

PAHs have been recognized as one of the most common organic pollutants. The six PAHs studied in this paper were pointed out to be the most widespread contaminants in nature, and they are the major components in oilfields [29,30]. The total concentrations of PAHs in the mean value were 31.4 ng/mL and 48.6 ng/mL in the plasma in the pre- and post-workshift, respectively. Compared to the level of PAHs in human blood in previous studies, the concentration of ∑_6_ PAHs was much higher than the median concentration measured in the American student’s plasma (0.281 ng/mL) [27], the mean concentration detected in the umbilical cord serum of pregnant women in the Shengsi Islands of China (0.80 ng/mL) [31], and the average PAH level of infertile men in their whole blood in a recent study (18.44 ng/mL) [15] (Table S4). Among the individual compounds, Phe was the most abundant PAH, with a median concentration of 13.3 ng/mL (pre-workshift) and 22.1 ng/mL (post-workshift), showing a dominance in PAH exposure in the study population. As reported by Huan Meng et al., Phe is a representative PAH that is present in crude oil [32]. To better identify the source of PAHs in plasma, PAH diagnostic ratios, which have been widely used in previous studies, were applied to the data analysis of PAHs in serum [33]. According to Yunker et al., a Fla/(Fla and Pyr) ratio below 0.4 implies a petroleum source, and a ratio above 0.5 indicates a pyrogenic source, in other words, a combustion source of biomass (such as coal combustion, grass, or wood). When the ratio ranges between 0.4 and 0.5, it indicates petroleum combustion, e.g., liquid fossil fuel and vehicle exhaust [34,35]. In this study, the Fla/(Fla and Pyr) ratio is 0.39 (pre-exposure) and 0.44 (post-exposure). The study shows that the petroleum source (derived from liquid petroleum before combustion) and petroleum combustion could be two major sources of PAHs. This finding could help better understand the mechanistic pathways and different source contributors, which could provide a scientific basis for future risk management and ease the stress of diseases caused by exposure to PAHs.

The mean values of the total concentrations of MPAHs and OPAHs in pre-workshift were 2.7 ng/mL and 7.2 ng/mL, respectively, and in post-workshift, they were 4.5 ng/mL and 8.7 ng/mL, respectively. So far, there are few data on the occurrence of MPAHs and OPAHs in human blood at home and aboard; hence, our understanding of exposure levels is limited. It is known that environmental exposure is one of the main sources of human exposure to PAHs. A recent study reported that in the soils of an industrial area in semi-arid Uzbekistan, PAH derivative (MPAHs and OPAH) levels were lower than those of their corresponding parent PAHs [36]. Another study showed that the concentrations of the OPAHs in the atmospheric particle phase were lower than their corresponding PAHs [37]. The results of these studies are different from ours. In our study, the concentration levels of MPAHs and OPAHs were higher than the parent PAHs. It is reported that PAH derivatives, similarly to their parent compounds, may originate from petrogenic or pyrogenic sources [11], which is a factor contributing to PAH derivatives in plasma. In addition, PAH derivatives are gradually generated from PAHs through a transformation in the metabolism of their parent PAHs in the human body [12,16]. The transformation from PAHs to PAH derivatives in human organisms results in the consumption of PAHs and the generation of PAH derivatives, due to which the concentration of MPAHs and OPAHs is higher than their parent PAHs. Though we do not know all about the toxicity of MPAHs and OPAHs, early evidence indicates that the developmental toxicity of OPAHs could be the same or even higher than that of their parent PAH [10]. Though environmental and toxicological concerns over PAH derivatives are increasing, research on these compounds remains limited. Therefore, there is an urgent need to study the impact of PAH derivatives on humans.

Over the past few years, there has been increasing interest in the effects of pollutant exposure on epigenetics, especially ROS-mediated methylation patterns. A few studies have fully demonstrated the relationship between PAH exposure and DNA methylation in organisms. A negative correlation between PAH concentration and DNA methylation of the interleukin 12 and p53 gene promoters was observed in Mexican brickmakers [20]. For zebrafish, when the DNA methylation level at the promoter regions of pdx-1 is increased, pancreatic endocrine development can be impaired by low-dose OPAH embryonic exposure [38]. The growing evidence suggests that mtDNA is sensitive to the damage caused by exogenous reactive oxygen sources [39,40,41]. Due to inefficient protective histones and the DNA repair system of mitochondria [42], mtDNA is especially vulnerable to ROS [43].

In the present study, using the bisulfite pyrosequencing approach, subtle methylation level differences in specific regions of the mitochondrial genome were observed. The level of mtDNA methylation increased in the post-workshift, among which the *MT-COX1* and *MT-COX2* methylation levels with the post-workshift were significantly (*p* < 0.05) higher than those of the pre-workshift. Through the study on the exposure–response relationship of PAHs, MPAHs, and OPAHs with mtDNA methylation in the plasma of oilfield workers, we found that the exposure–response relationship between PAHs and mtDNA methylation in the plasma of workers was significant (*p* < 0.05); the exposure to Ant could induce the up-regulation of the methylation of *MT-COX1* (*β* = 0.831, SD = 0.105, *p* < 0.05); and the exposure to Flo and Phe could induce the up-regulation of the methylation of *MT-COX3* (*β* = 0.115, SD = 0.042, *p* < 0.05 and *β* = 0.036, SD = 0.015, *p* < 0.05; respectively). Though exposure burden was also detected for other PAH compounds, such as Dibf and Phy, no significant correlations were found between these compounds and mtDNA methylation. Since the mechanisms of the influence of environmental exposures on mtDNA epigenetic modifications has not been well explored, further studies should be conducted in the future.

mtDNA methylation is essential for the genome stability, gene expression, and function of mitochondria and can play a vital role in the pathophysiological processes of many diseases [39]. The increase in mtDNA methylation may induce dysfunctional mitochondria [28]. Dysfunctional mitochondria can cause a variety of diseases, such as diabetes and neurodegeneration [44,45]. It is known that dysfunctional COX complexes are the most severe mitochondrial diseases [46]. *COX* is a component of the respiratory electron transport chain, and the subunits 1 to 3 (*MT-COX1*, *MT-COX2*, and *MT-COX3*) form the functional core of complex IV. Genetic mutations in *COX* genes can cause severe metabolic diseases [46] that mainly affect high-energy-demanding tissues, such as the heart [39,47,48,49]. Previous studies have reported that the mtDNA methylations of cardiovascular disease (CVD) patients are significantly higher than those of healthy controls in *MT-COX1*, *MT-COX2*, and *MT-COX3* [28]. In addition, platelets are related to the formation of blood clots, and low platelet *COX* activity can lead to abnormal clotting, which may cause heart attacks, stroke, and other CVD risks. Platelet dysfunction is also involved in hyperaggregation and activation, which may also pose risks for CVD [50]. Therefore, inflammatory processes and disorders can be caused by the disruption of *COX* expression by epigenetic mechanisms [40]. This report showed that PAHs can induce the up-regulation of mtDNA methylation and demonstrated that mtDNA methylation is a potential sensitive biomarker to be used to measure the effects of PAHs and their effects on human health. Novel biomarkers of mtDNA methylation can help modify exposure to the risk factors of POPs and contribute to cancer prevention.

This study is unique in the way it was conducted. It was a thorough study in which PAH, MPAH, and OPAH exposure and mtDNA methylation were evaluated, and the effect of pollutants on mtDNA methylation’s alteration was analyzed. mtDNA is expressed as matrilineal inheritance, and studies on its structure and proportions can better reflect the genetic characteristics of populations. In addition, PAHs, MPAHs, OPAHs, and mtDNA methylation were detected using GC-MS and pyrosequencing, respectively, [27,51], which are considered to be precise, accurate, sensitive, and reproducible. However, there are limitations to this study too. In this present study, we conducted an analysis of mtDNA methylation in three circumscribed regions of the mitochondrial genome, and PAHs and their derivatives may also be associated with DNA methylation in other regions of the mtDNA. Second, other pollutants such as heavy metals could induce body global DNA hypomethylation [19,52]. In addition, it’s known that PAHs can be ingested with food [53,54]. The diet of oilfield workers changed over the course of the workshift, which might induce alterations in the concentration of PAHs in the blood. However, details of the diet were not collected during the survey, which was also a limitation of our study. Whether they can induce mtDNA methylation together with PAHs needs further investigation.

## 5. Conclusions

In conclusion, we found an exposure–response relationship between polycyclic aromatic hydrocarbons and mtDNA methylation. The result showed that Ant could induce the up-regulation of the methylation of *MT-COX1*, and Flo and Phe could induce the up-regulation of *MT-COX3*, respectively. mtDNA methylation might be a potential biomarker for the assessment of health risks from. Future studies with larger sample sizes, repeated sampling, and analyses of additional epigenetic and exposure biomarkers are needed.

## Figures and Tables

**Table 1:** PCR and pyrosequencing primer sequence.

Gene Name	Primer	Sequence
*MT-COX1*	Forward primer (5′ to 3′)	TATTAATTGGTTTTTTAGGGTTTAT
	Reverse biotin primer (5′ to 3′)	CAACAAATCATTTCATATTACTTCC
	Sequencing primer (5′ to 3′)	TATTTATAGTAGGAAT
*MT-COX2*	Forward primer (5′ to 3′)	TTTATGAGTTGTTTTTATATTAGGTTTAAA
	Reverse biotin primer (5′ to 3′)	ACTCCACAAATTTCAAAACATTAAC
	Sequencing primer (5′ to 3′)	TAAAAATAGATGTAAT
*MT-COX3*	Forward primer (5′ to 3′)	TATATTATTTGTTTAAAAAGGTTTT
	Reverse biotin primer (5′ to 3′)	AATAAAAAACTCAAAAAAATCCTAC
	Sequencing primer (5′ to 3′)	TATATTATTTGTTTAAAAAGGTTTT

**Table 2:** Individual exposure concentration of PAHs in pre- and post-workshift.

PAHs	Pre-Workshift			Post-Workshift			*p*-Value
	Mean	Median (P_25_–P_75_)	Detection Frequency	Mean	Median, (P_25_–P_75_)	Detection Frequency	
Nap	0	0	0%	1.8	1.7 (1.4, 2.1)	44.4%	/
Flo	4.1	3.9 (2.6, 5.7)	94.4%	6.6	6.0 (3.7, 9.0)	83.3%	0.0304 *
Phe	14.6	13.3 (11.3, 19.2)	100%	23.4	22.1 (13.4, 29.1)	83.3%	0.0077 *
Ant	1.7	1.6 (1.5, 2.0)	44.4%	2.7	2.9 (1.9, 3.2)	55.5%	0.0013 *
Flu	3.3	3.5 (2.2, 3.8)	94.4%	6.0	5.2 (4.2, 7.7)	72.2%	0.0430 *
Pyr	7.7	6.1 (3.97, 7.8)	100%	8.2	7.6 (3.6, 11.6)	94.4%	0.9617
Nap-1	1.3	1.3 (1.3, 1.3)	5.5%	2.0	1.8 (1.4, 2.3)	33.3%	/
Nap-2	1.4	1.1 (1.1, 1.6)	44.4%	2.5	2.2 (1.7, 2.9)	61.1%	0.2151
Dibf	7.2	5.4 (3.3, 8.0)	94.4%	8.7	6.0 (4.9, 14.1)	83.3%	0.8130
The total of all components	41.3	35.2 (24.0, 48.5)	/	61.8	44.0 (19.7, 66.8)	/	0.2948

Among all contaminants, NAP and NAP-1 were not available for statistical analysis due to their low detection frequency. The *p*-value was calculated by the group square difference in random area for post hoc comparisons. The model analysis results adjusted for age, BMI, and length of service. * *p* < 0.05, indicating a statistical significance.

**Table 3:** The associations of mtDNA methylation with PAHs and PAH derivative exposure concentrations in human plasma.

Variables	*MT−COX1*			*MT−COX2*			*MT−COX3*		
	*β*	SD	*p*-Value	*β*	SD	*p*-Value	*β*	SD	*p*-Value
Nap−2	0.688	0.555	0.243	0.680	0.817	0.424	0.089	0.142	0.213
Dibf	0.008	0.095	0.934	−0.255	0.147	0.105	0.058	0.028	0.545
Flo	0.126	0.185	0.508	−0.083	0.251	0.745	0.115	0.042	0.016 *
Phe	0.088	0.063	0.188	0.091	0.092	0.337	0.036	0.015	0.037 *
Ant	0.831	0.105	0.000 *	1.893	1.364	0.199	0.192	0.232	0.430
Flu	0.270	0.208	0.215	0.365	0.342	0.303	0.064	0.070	0.372
Pyr	−0.022	0.067	0.744	0.046	0.122	0.711	0.018	0.023	0.457
The total of all components	0.011	0.020	0.590	0.015	0.027	0.606	0.006	0.005	0.245

The model analysis results adjusted for age, BMI, and length of service. Regression coefficient *β* represents the estimated change in mtDNA methylation with one-unit (1 ng/mL) increments in pollutant concentration. The *p*-value calculated by mixed-effect models. * *p* < 0.05, indicating a statistical significance.

## Data Availability

The authors confirm that the data supporting the findings of this study are available within the article (and/or) its Supplementary Materials.

## Supplementary Materials

The following are available online at https://www.mdpi.com/article/10.3390/toxics11050466/s1, Table S1: Dwelling times of the oilfield workers; Table S2: The monitored ions and CAS number of 9 analytes and surrogate standards; Table S3: The methylation levels of *MT-COX1*, *MT-COX2*, and *MT-COX3* in pre- and post-workshift; Table S4: The comparison of PAHs contributions of human blood in the recent literature.
