# Supplementary material for: The Levels of Polycyclic Aromatic Hydrocarbons and Their Derivatives in Plasma and Their Effect on Mitochondrial DNA Methylation in the Oilfield Workers
Source: Toxics. 2023 May 17;11(5):466. doi: 10.3390/toxics11050466 (PMC10220707; doi:10.3390/toxics11050466)
Supplement: Supplementary file 1 [file toxics-11-00466-s001.zip › toxics-2393152-supplementary.pdf]

Supporting Information

Table S1 Dwelling times of the oilfield workers.

Table S2 The monitored ions and CAS number of 9 analytes and surrogate standards.

Table S3 The Methylation levels of *MT-COX1*, *MT-COX2* and *MT-COX3* in pre- and post-workshift.

Table S4 The comparison of PAHs contributions of human blood in the recent literature.

Supporting Information

Table S1. Dwelling times of the oilfield workers

ID	Time of departure	Return time	Dwelling times (day)
1	2015-12-23	2016-01-20	28
2	2016-01-06	2016-02-03	28
3	2015-12-30	2016-01-28	29
4	2015-12-23	2016-01-14	22
5	2015-12-09	2016-01-07	29
6	2016-01-27	2016-02-24	28
7	2015-12-16	2016-01-07	22
8	2016-01-13	2016-02-04	22
9	2015-12-23	2016-01-21	29
10	2016-02-03	2016-03-02	28
11	2016-01-27	2016-02-24	28
12	2015-12-30	2016-01-28	29
13	2016-01-06	2016-02-02	27
14	2015-12-09	2016-01-07	29
15	2015-12-30	2016-01-28	29
16	2016-01-20	2016-02-04	15
17	2015-12-22	2016-01-28	37
18	2016-01-13	2016-02-11	29
19	2015-12-30	2016-02-02	34

Table S2. The monitored ions and CAS number of 9 analytes and surrogate standards

Chemical compound	CAS number	quantifier ion	qualifier ion
Naphthalene (Nap)	91-20-3	128	127.00-129.00
Fluorene (Flo)	86-73-7	166	165.00-164.00
Phenanthrene (Phe)	85-01-8	178	176.00-152.00
Anthracene (Ant)	120-12-7	178	176.00-179.00
Fluoranthene (Flu)	206-44-0	202	200.00-203.00
Pyrene (Pyr)	129-00-0	202	200.00-201.00
Naphthalene, 1-methyl- (Nap-1)	90-12-0	142	141.00-115.00
Naphthalene, 2-methyl- (Nap-2)	91-57-6	142	141.00-115.00
Dibenzofuran (Dibf)	132-64-9	168	139.00-169.00
Phenanthrene-D_{10} (Phe-D_{10})	1517-22-2	188	184.00-160.00
Chrysene-D_{12} (Chr-D_{12})	1719-03-5	240	236.00-241.00

Table S3. The Methylation levels of *MT-COX1, MT-COX2* and *MT-COX3* in pre- and post-workshift

Gene name	Pre- workshift (median, P_{25}-P_{75})	Post- workshift (median, P_{25}-P_{75})	*P*-value
MT-COX1	13.18（10.55,14.82)	16 (13.43,16.97)	0.005*
MT-COX2	14.84 (11.51,16.67）	20.55 (17.32,23.31)	0.004*
MT-COX3	3.67（3.08,4.91)	4.69（3.94,5.10)	0.130

[a]The p-value calculated by the group square difference of random area for post hoc comparisons. *P*<0.05, indicating a statistical significance

Table S4. The comparison of PAHs contributions of human blood in the recent literature

study		Reference	Nap(ng/ml)	Flo	Phe	Ant	Flu	Pyr	Σ_6PAHs
This study, plasma	Pre-exposure	/	ND	4.1	14.6	1.7	3.3	6.7	31.4
	Post-exposure	/	1.8	6.6	23.4	2.7	6.0	8.2	48.6
Chinese women, umbilical cord serum		(Yin et al., 2017)	0.16	0.01	0.04	0.03	0.07	0.49	0.80
infertile men in China, whole blood		(Song et al., 2013)	1.47	1.90	1.18	1.81	11.84	0.24	18.44
American students, plasma		(Pleil et al., 2010)	0.183	0.017	0.042	0.012	0.014	0.013	0.281
